# Nerve Disease

**Published:** 1894-03-03

**Authors:** 


### Medical Progress.

### NERVE DISEASES.

*Allachæsthesia.*—Professor Grainger Stewart[1] reports an unique case, to which he gives the foregoing name. The subject was a middle-aged man with continuous spontaneous ankle-clonus on the right side and choreiform movements of the right hand and left foot, knee-jerk exaggerated on both sides, artificial ankle-clonus obtainable on both sides, and a peculiar mode of walking, this consisting of three steps being taken towards the right, followed by three steps towards the left, caused by exaggerated adduction of the thigh occurring at each second step of the three-step movement. These constituted the sole objective phenomena. The peculiarity of the case lies in the nature of the subjective sensory phenomena, confined to the left half of the body. Here all kinds of skin impressions were perceived readily, and with normal acuteness, but they were incorrectly localised. This erroneous localisation did not vary, but in the arm and leg constantly showed that impressions of touch, of pain, of temperature, applied, say, over the middle third of the radius, was perceived as if applied over the middle third of the ulna. The left half of the trunk showed the same peculiarity, but above the clavicle there was no abnormality. In both arm and leg there were, however, small areas in which localisation was accurate, these being a strip in the middle of the flexor and of the extensor surfaces. No abnormality could be discovered in the electrical reactions of nerves or muscles. Malingering being thought possible, close watch was kept upon the patient for three months, without any alteration in either the objective or subjective phenomena being discoverable. The condition which approaches most closely this case is that described by Obersteiner under the name of allocheiria, in which, however, the touch applied on one side is simply referred to the corresponding spot upon the other side of the body, and it may affect one kind of sensory impression, or all the varieties, including sight and hearing. Even reflex movements and electrical stimulation may be transferred to the other side of the body in the manifestation of their effects. If the true seat of the lesion were situated in the spinal cord or higher sensory tracts, as is supposed to be the case in allocheiria, one would expect also altered sensibility of some kind, which in Grainger Stewart's case did not exist. He thinks the condition functional—that the fault lies not so much in the conducting fibres as in a morbid action of the sensory centres.

*Chorea.*—Osler[2] records the history of two families in which hereditary (Huntingdon's) chorea occurred. In one of the families of the first generation two out of eleven were affected; of these two persons, the male died childless and demented, the symptoms beginning at the age of forty; the female, in whom the symptoms began before the age of forty, died at sixty-five mentally almost sound. She had five children who were all affected, the two males commencing at the ages of sixty-two and forty-two respectively, and three females, who became affected at the ages of forty, thirty-four, and forty-two respectively. Of the third generation, however, none are affected as yet, although several are over thirty-five years of age. A post-mortem examination being made upon a female example, who died at fifty-one years of age, the arteries of the brain were found to be thickened with occasional patches of hyaline degeneration, and fatty degeneration was found in the smaller arterioles. Here and there the perivascular spaces were large and contained leucocytes. The ganglion cells showed very slight changes, vacuolation and pigmentation; there was general wasting of the convolutions, which were firmer than usual, but showed few microscopic changes.

Osler classifies chronic chorea as follows: (1) *Chorea of*

*infants*, appearing at birth, or within the first three years of life—(*a*) majority of cases are examples of spastic diplegia with choreiform or athetoid movements; (*b*) typical choreic movements without spasm; (*c*) a few cases of chronic progressive chorea with dementia begin in infancy. (2) *Non-hereditary chronic chorea*, beginning at any time from childhood onwards and leading to dementia after a variable period. Resembles Huntingdon's form. (3) *Huntingdon's hereditary chorea*, characterised by family groups, late onset, psychical disturbances, progressive course, and fatal termination. (4) *Chronic chorea minor*, which may go on for months or years and end in recovery. *Tic convulsif*, with typical choreiform movements, may be confounded with chorea minor, but convulsive tic can generally be diagnosed by the evidence of fixed ideas, coprolalia, &c.

Dr. J. W. Russell[3] records two cases of hereditary chorea occurring in twins. Of these certainly the father, and possibly the paternal grandmother, were choreic. The patients, aged thirty-four, had been choreic each about seven years. The movements were very marked, aggravated by observation. The knee-jerks were exaggerated (Gowers describes them as normal), and ankle-clonus could be usually obtained. In both cases there was little deterioration of the mental faculties, although one seemed slightly deficient in memory.

*Treatment*.—Deydier[4] publishes five cases of chorea (Sydenham's) treated by injection of orchitic extract. One case was cured by seven injections; one was quite rebellious to the treatment; in the other three cases cure was obtained in three or four weeks. Eskridge[5] relies mainly on antipyrin at first. When the violence of the movements has largely subsided he gives Fowler's solution, beginning with one-drop doses thrice daily, increasing the dose one drop each day. He thinks tolerance is reached generally when the dose has reached six or seven drops. Dr. Bertram Rogers,[6] like most English physicians, considers that arsenic must be given in full doses in order for most benefit to be obtained.

*Disease of the Corpora Quadrigemina*.—Dr. F. Taylor[7] described at the Medical Society of London a case occurring in a boy of four years of age. Drooping eyelids, loss of strength, then staggering gait and ataxy of upper and lower limbs were the earlier symptoms. Double external ophthalmoplegia, double ptosis more marked on the right side, lateral nystagmus, pupils slightly and unequally dilated, no optic neuritis (although the child could not see) were later developments. He became unconscious, breathing assumed Cheyne-Stokes' character, and finally swallowing became impossible. Death took place six months after the first symptom. Cerebellum was found to be healthy, but the corpora quadrigemina the seat of glio-sarcomatous growth. The case illustrated completely Nothnagel's formula for disease of, or in the neighbourhood of the corpora quadrigemina: "An unsteady, reeling gait, especially if appearing as the first symptom, with ophthalmoplegia existing in both eyes, not quite symmetrically, nor implicating all the muscles in equal degrees."

*Paralysis Agitans*.—Ketscher[8] has investigated the pathological anatomy of three undoubted cases. The nervous system, central and peripheral, was found to exhibit changes in each case. The nervous structures showed atrophy, the ganglion cells were deeply pigmented and altered in form, the nerve fibres both in the peripheral nerves and spinal cord were degenerated and had in some instances disappeared, while the muscular fibres were also atrophied and degenerated. The neuroglia was thickened, especially around the vessels, and mostly in the posterior and lateral columns. The vessels also were altered, their walls thickened, and there were miliary aneurisms and small hæmorrhages present. Ketscher concludes, with Borgherini and others, that paralysis agitans is only the expression of an extreme and premature senility of the nervous system, and that the primary changes are in the vessels, those in the nervous structures being secondary.

*Plumbism*.—Ebstein[9] discovered lead in the brain of a man who died from interstitial nephritis, and had had lead-colic eight years previously. There was no blue line on the gums, and no lead was found in the muscles. He points out the interest of this observation in relation to the etiology of encephalopathia saturnina, which has been attributed to the presence of lead in the brain. In the present case there was no history of any encephalopathia saturnina, whilst in one of Oliver's cases of this affection no lead could be detected after death in the brain.

The committee appointed by the Home Secretary to inquire into the conditions of manufacture of the various branches of the lead industry has issued a valuable report. Although dealing with the medical aspect of the question, it does not directly bear upon the neurological element.

*Syphilis of the Spinal Cord*.—At the Medical Society of Berlin, November 15th, Gerhardt[10], speaking upon the subject of syphilis of the cord, stated it affects the meninges and vessels, but very seldom the cord tissue itself primarily. Syphilis is a cause of many cases of disease of nerve roots. The great majority of the patients present the group of symptoms described by Erb as characteristic of spastic spinal paralysis of syphilitic origin, which commences as a slowly advancing hemiplegia, the sphincters being more or less involved. Gerhardt finds a history of syphilis in one half of his cases of tabes, A. Fraenkel the same, Mendel and Senator from 70 to 75 per cent. Virchow, on the other hand, considers that no connection between the two morbid conditions has been proved to exist.

*Progressive Muscular Atrophy*.—Græme Hammond[11] records the symptoms during life, and the microscopical appearances in the cords, belonging to two cases of progressive muscular atrophy. The pyramidal tracts, both crossed and uncrossed, were sclerosed, with no symptoms of such in one case during life. If progressive muscular atrophy and amyotrophic lateral sclerosis be distinct in their origins, it can only be that the former begins in the anterior cornua, and degeneration of the pyramidal tracts occurs subsequently; in the latter, degeneration evidently begins in the pyramidal tracts, and spreads to the anterior cornua. Pathologically the diseases are identical, in their later stages at least, if not originally.

*Operation for Disease of Cauda Equina*.—Dr, J. E. Shaw and Mr. J. P. Bush[12] record a case of disease of the cauda equina which was diagnosed, and operated upon with some relief to the excruciating pains which the patient suffered in his legs and thighs.

*Acute Ascending Myelitis.*—Dr. W. R. Gowers[13] records a case of fatal acute ascending myelitis in a young man, due apparently to toxic infection by the gonococcus.

[1] Brit. Med. Journ., Jan. 6, 1894. [2] Journ. Nerv. and Ment. Dis., Feb., 1893, quoted in Med. Chron., Jan., 1894. [3] Birm. Med. Rev., Jan., 1894. [4] Lyon Méd., No. 16, 1893, quoted in Amer. Journ. Med. Sci., Jan., 1894. [5] Med. News, Sept. 30, 1893. [6] The Hospital, Dec. 30, 1893. [7] Practitioner, Jan., 1894. [8] Quoted in N. York Med. Record, Sept. 1, 1893. [9] Virchow's Archiv., quoted in Ep. B. M. J., Jan. 6, 1894. [10] Med. Week, Dec. 1, 1893. [11] N. York Med. Journ., Jan. 6, 1894. [12] Bristol Medico-chirurg. Journ., Sept., 1893. [13] Clinical Journal, Dec. 20, 1893.

(*To be continued.*)

